# Differentiating apathy and depression across neurodegenerative disorders: sex‐specific associations with severity and location of white matter hyperintensities

**DOI:** 10.1002/alz70857_106627

**Published:** 2025-12-25

**Authors:** Almira Siddiqui, Joel Ramirez, Shankar Tumati, Nathan Herrmann, Dallas Seitz, Krista L Lanctôt

**Affiliations:** ^1^ University of Toronto, Toronto, ON, Canada; ^2^ Sunnybrook Research Institute, Toronto, ON, Canada; ^3^ Sunnybrook Health Sciences Centre, Toronto, ON, Canada; ^4^ Hotchkiss Brain Institute, University of Calgary, Calgary, AB, Canada

## Abstract

**Background:**

Apathy and depression are overlapping symptoms in neurocognitive disorders (NCDs). White matter hyperintensities (WMH), indicative of microvascular pathology, have been associated with apathy and depression individually. Less is known about these symptoms’ spatial distribution and sex‐specific patterns, particularly when they co‐occur. It was hypothesized that individuals with comorbid apathy and depression (A+D) would exhibit higher WMH severity.

**Method:**

Data from the COMPASS‐ND longitudinal cohort of participants with various NCDs were categorized into four groups based on the presence, absence, or comorbidity of apathy and depressive symptoms: apathy only (AO), depressive symptoms only (DO), A+D, and neither apathy nor depression (NAD). Apathy and depressive symptom presence was assessed using the Neuropsychiatric Inventory Questionnaire, while WMH severity was evaluated with the Fazekas scale (graded 0 to 3) in periventricular (PV) and subcortical (SC) regions. Ordinal regression analyses, adjusted for age, sex, and Montreal Cognitive Assessment (MoCA) scores, examined the relationships between group (AO, DO, A+D, NAD) and regional WMH severity. Separate analyses explored potential sex‐specific patterns.

**Result:**

The 803 participants were composed of various NCDs, including mild cognitive impairment (MCI) (*n* = 217), vascular MCI (*n* = 135), Alzheimer's disease dementia (*n* = 72), and other diagnoses (e.g., mixed dementias, vascular dementia, Parkinson's disease, Lewy body dementia, frontotemporal dementia, etc) (*n* = 377). Participants had a mean (SD) MoCA score of 22.8 (4.7) (range 4‐30) and a mean age of 72.1 (7.6) years, with 55.8% males. Groups included AO (*n* = 106, 13.2%), DO (*n* = 120, 15%), A+D (*n* = 135, 16.8%), or NAD (*n* = 442, 55%). Neither PVWMH nor SCWMH were associated with either AO or DO. A+D was associated with higher PVWMH severity (β=0.508, SE=0.201, *p* = 0.01) but not SCWMH severity. In sex‐specific analyses, males with A+D exhibited higher PVWMH severity (β=0.765, SE=0.242, *p* = 0.002), whereas no significant associations were found between groups and WMH severity in females.

**Conclusion:**

This study demonstrated a positive relationship between A+D and PVWMH, particularly in males, while no associations were present in females or with separate symptoms. These findings support the need for regional and sex‐specific analyses of the relationship between WMH and comorbid apathy and depression across NCDs to improve their understanding and potentially inform treatment approaches.

